# Dietary Zinc Deficiency Affects Blood Linoleic Acid: Dihomo-γ-linolenic Acid (LA:DGLA) Ratio; a Sensitive Physiological Marker of Zinc Status *in Vivo* (*Gallus gallus*)

**DOI:** 10.3390/nu6031164

**Published:** 2014-03-20

**Authors:** Spenser Reed, Xia Qin, Rinat Ran-Ressler, James Thomas Brenna, Raymond P. Glahn, Elad Tako

**Affiliations:** 1 USDA-ARS, Robert Holley Center for Agriculture & Health, Ithaca, NY 14853, USA; E-Mails: smr292@cornell.edu (S.R.); raymond.glahn@ars.usda.gov (R.P.G.); 2 Division of Nutritional Sciences, Cornell University, Ithaca, NY 14853, USA; E-Mails: Rinat.Ran-Ressler@rd.nestle.com (R.R.-R.); jtb4@cornell.edu (J.T.B.); 3 Department of Food Science, Cornell University, Ithaca, NY 14853, USA; E-Mail: xq37@cornell.edu

**Keywords:** zinc, zinc deficiency, zinc biomarker, broiler chicken, Δ^6^ desaturase, delta-6 desaturase, red blood cell fatty acids

## Abstract

Zinc is a vital micronutrient used for over 300 enzymatic reactions and multiple biochemical and structural processes in the body. To date, sensitive and specific biological markers of zinc status are still needed. The aim of this study was to evaluate *Gallus gallus* as an *in vivo* model in the context of assessing the sensitivity of a previously unexplored potential zinc biomarker, the erythrocyte linoleic acid: dihomo-γ-linolenic acid (LA:DGLA) ratio. Diets identical in composition were formulated and two groups of birds (*n* = 12) were randomly separated upon hatching into two diets, Zn(+) (zinc adequate control, 42.3 μg/g zinc), and Zn(−) (zinc deficient, 2.5 μg/g zinc). Dietary zinc intake, body weight, serum zinc, and the erythrocyte fatty acid profile were measured weekly. At the conclusion of the study, tissues were collected for gene expression analysis. Body weight, feed consumption, zinc intake, and serum zinc were higher in the Zn(+) control *versus* Zn(−) group (*p* < 0.05). Hepatic TNF-α, IL-1β, and IL-6 gene expression were higher in the Zn(+) control group (*p* < 0.05), and hepatic Δ^6^ desaturase was significantly higher in the Zn(+) group (*p* < 0.001). The LA:DGLA ratio was significantly elevated in the Zn(−) group compared to the Zn(+) group (22.6 ± 0.5 and 18.5 ± 0.5, % w/w, respectively, *p* < 0.001). This study suggests erythrocyte LA:DGLA is able to differentiate zinc status between zinc adequate and zinc deficient birds, and may be a sensitive biomarker to assess dietary zinc manipulation.

## 1. Introduction

Zinc is one of the most abundant trace minerals in cells, and is essential for growth and development of nearly all organisms [1]. With 1.5–2.5 g of zinc present in the average adult [2], zinc is second only to iron in total body trace mineral content. It is found primarily in tissues such as the brain, kidneys, pancreas and liver with smaller concentrations in hair, skin and fingernails [3]. Zinc is vital for numerous physiological and metabolic processes, such as acting as an antioxidant and cofactor in over 300 zinc metalloenzymes [4,5]. Zinc functions in the regulation of an extensive variety of genes such as those involved in nucleic acid metabolism [6], cell signaling [7,8], and apoptosis [9]. Zinc also plays an integral role in immune system functioning [10,11,12,13]. Since there is no readily-accessible storage form of zinc, regulation is highly efficient [14,15,16]. In humans, tight regulation is accomplished by the control of zinc absorption and transport to and from the small intestine via two families of transmembrane proteins [17,18,19,20,21], solute carrier 30A (*Slc30a*, ZnT) and *Slc39a* members (ZIP). As was previously demonstrated, expression of several of these proteins is usually upregulated in zinc deficiency conditions [17].

However, despite an increasing understanding of zinc homeostasis, the paucity of sensitive zinc biomarkers, as well as a representative animal model in which to test them, has made assessment of zinc deficiency difficult to both quantify and categorize. Although whole blood, plasma, and urine zinc decrease in severe zinc deficiency, accurate assessment of zinc status, especially in mild to moderate deficiency, is difficult as studies with these biomarkers are often contradictory and inconsistent. In their recent meta-analysis on biological indicators of zinc status, Lowe *et al.* concluded plasma, serum, urinary, and hair zinc were the only effective biological indicators out of 32 potential biomarkers from 46 publications in humans [22]. Previous studies have shown plasma and serum zinc to be insensitive indicators of zinc status [23,24,25], although some studies demonstrate they respond to both depletion and repletion of zinc [26,27]. Erythrocyte zinc is often used to evaluate zinc status, although this biomarker has been shown to be both responsive [25] and non-responsive [26] to zinc depletion. Further, purported biomarkers such as hair [26], urinary [25], and fecal zinc [22] have shown mixed efficacy as sensitive biomarkers of zinc status during dietary intervention; these discrepancies may be independent of differences in experimental protocol. The need to develop additional robust indicators of zinc status and expound upon the already known clinical markers, for which limited data of reliability exists, is evident.

The broiler chicken (*Gallus gallus*) matures relatively quickly and is sensitive to dietary manipulation of various micronutrients such as zinc [28,29,30,31]. As was previously demonstrated [32,33,34,35], the broiler chicken is a responsive model in which to test dietary iron bioavailability. Further studies have shown the broiler chicken highly sensitive to dietary zinc manipulation [28,29,30,31,32,33,34,35]. Also, it was previously demonstrated that birds have a similar membrane fatty acid composition to mammals [36], which makes it a potentially-ideal animal model to study essential fatty acid (EFA) accumulation in relation to mineral nutrition.

Using this animal model, we identified and implemented a previously unexplored biomarker of zinc status pertaining to erythrocyte Δ^6^ desaturation. The physiology of membrane fatty acid accumulation in relation to zinc homeostasis has been the subject of little investigation in the literature, even though zinc plays a crucial role as a cofactor for the desaturase enzymes [37,38]. Initial studies proposed a role for zinc and EFAs in swine parakeratosis [39]. Bettger *et al.* later suggested a physiological connection between zinc and EFAs, as zinc deficiency increased proportions of arachidonic acid (20:4ω6) [40]. Horrobin *et al.* postulated that desaturase enzymes require zinc as a cofactor for proper functioning [38]. Desaturase enzymes have both a requirement for zinc and a relatively low binding constant [41,42], thus their activity is quite sensitive to early-stage zinc deficiency. What ensues is a disturbed ratio of their substrates and products, in this case linoleic acid (LA, 18:2ω6) and dihomo-γ-linolenic acid (DGLA, 20:3ω6), respectively. The Δ^6^-catalyzed step required for conversion of 18:2ω6 to 20:3ω6 is usually the highest flux pathway [43], so an elevation in the 18:2ω6:20:3ω6 ratio may be a sensitive marker for zinc deficiency.

**Figure 1:**
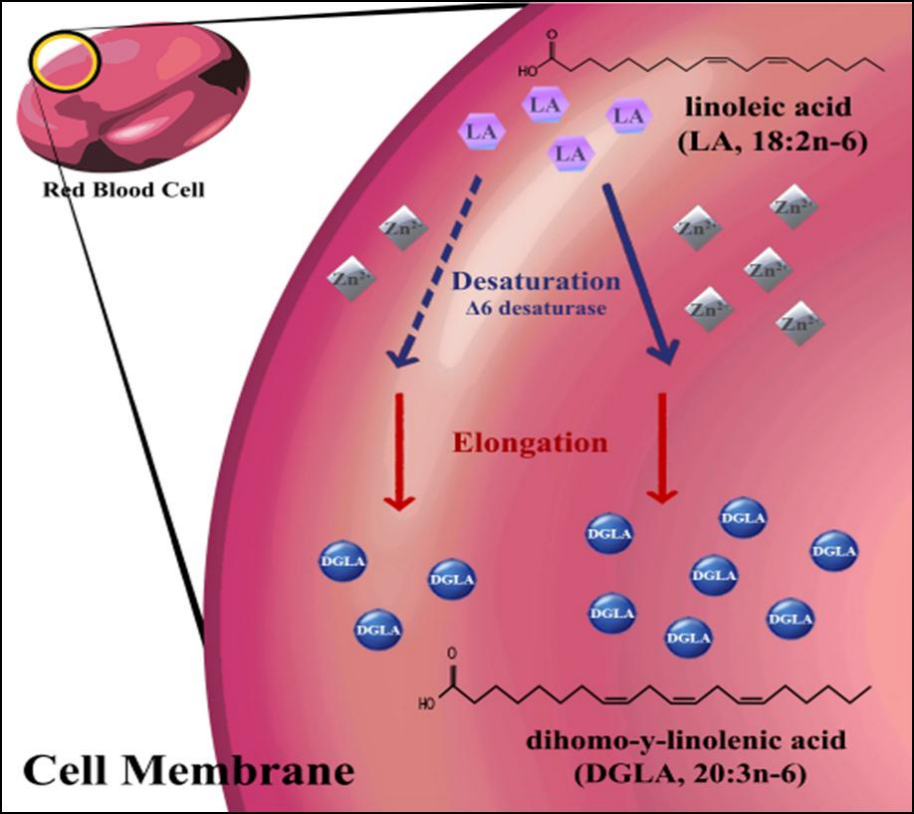
A truncated schematic of the LA to DGLA fatty acid pathway within the erythrocyte membrane. Lack of dietary zinc (broken line), needed for Δ^6^ desaturase enzyme function, will impede conversion of reactant (LA) to product (DGLA) and will result in an increased ratio of LA to DGLA. This ratio may be a sensitive biomarker to identify endogenous zinc deficiency.

It is known [37] that, because of the zinc requirement of Δ^6^ desaturase, increasing dietary γ-linolenic acid (18:3ω6, a direct product of linoleic acid desaturation) may correct for the biological effects of zinc deficiency in terms of membrane EFA composition. Similarly, increasing dietary concentrations of LA may modify the LA:DGLA ratio independent of zinc status. Therefore, the knowledge of and/or controlling for dietary concentration of both fatty acids may be important in qualifying the specificity of the LA:DGLA biomarker. Thus, we investigated whether the ratio of 18:2ω6:20:3ω6 could be implemented as a sensitive biomarker of zinc status *in vivo* during the length of a controlled feeding trial. Also, hepatic mRNA gene expression for the Δ^6^ desaturase enzyme was measured. Figure 1 represents the role zinc plays in the rate-limiting desaturase step of the ω6 fatty acid pathway.

To provide context in determining the sensitivity of the LA:DGLA biomarker, we also assessed several relevant zinc-dependent factors, such as the expression of zinc transporter proteins (ZnT1, ZnT5, ZnT7, ZIP6, ZIP9, and DMT-1), immunomodulatory cytokines (TNF-α, IL-1β, IL-6), a transcription factor (NF-κB), zinc-dependent digestive (AP and SI) and metabolically-relevant (Na^+^K^+^ATPase and SGLT-1) enzymes, a zinc binding protein (MT4), as well as physiological markers (body weight, feather, nail, and serum zinc).

We hypothesize that the LA:DGLA biomarker assessed in this study will respond to changing levels of dietary zinc. Such a biomarker could be used to rapidly screen zinc status *in vivo*. Therefore, the primary objective of this study was to evaluate the response of the LA:DGLA ratio, as well as other indices of zinc nutriture, to dietary zinc deficiency in the *Gallus gallus* model. This study reports on the initial findings of the development and implementation of the LA:DGLA biomarker.

## 2. Experimental Section

### 2.1. Animals, Diets and Study Design

Forty-eight Cornish cross-fertile broiler eggs were obtained from a commercial hatchery (Moyer’s Chicks, Quakertown, PA, USA). The eggs were incubated under optimal conditions at the Cornell University Animal Science Poultry Farm incubator. Upon hatching (hatchability rate was 94%), chicks were randomly allocated into two treatment groups on the basis of body weight and gender (aimed to ensure equal distribution between groups, *n* = 12): (1) Zinc adequate control diet (Zn(+), 42.29 μg/g zinc); and (2) Zinc deficient diet (Zn(−), 2.55 μg/g zinc). Experimental diets differed only on the basis of supplemental zinc (as zinc carbonate). Table 1 represents the two diets. Table 2 represents the fatty acid composition of the two diets. Chicks were housed in a total-confinement building (one chick per 0.5 m^2^ metal cage). Birds were under indoor controlled temperatures and were provided 16 h of light. All birds were given *ad libitum* access to purified water and food. Feed intakes were measured daily (as from day one), and body weight was measured weekly.

**Table 1:** Composition of the experimental diets *.

Ingredient	Zn(+) Control Diet	Zn(−) Diet
**g/kg (by Formulation)**		
Egg whites	200	200
dl-Methionine	3	3
Cornstarch	318.2	318.2
Dyetrose	105	105
Dextrose	200.0	190.8
Cellulose	50	50
Corn oil	50	50
Salt mix (no Zn)	60	60
Vitamin mix	10	10
Biotin (1 mg/g)	1.8	1.8
Choline bitartrate	2	2
Zinc carbonate (5 mg/g)	9.2	−
Total (g)	1000	1000
**Concentrations of Selected Components** (means ± SEM, *n* = 5)		
Zinc concentration (ppm) **	42.29 ^a^ ± 0.25	2.55 ^b^ ± 0.02
Iron concentration (ppm)	98.75 ± 2.04	102.19 ± 5.21
Phytic acid ***	<dL	<dL

* Modified NRC [44] purified chicken diets were provided by Dyets Inc. (Bethlehem, PA, USA), (Zn(+), Zn adequate control diet: 135251, Zn(−), Zn deficient diet: 135252). ** Determination of zinc concentration is described in the materials and methods section. *** Determination of phytic acid in the diet is described in the Materials and Methods sections. Values are below the detection limit (dL). ^a,b^ Within a column, means without a common letter are significantly different (*p* < 0.05).

**Table 2:** Fatty acid composition of the Zn(+) control and Zn(−) diets *^,^**.

Fatty Acid	Zn(+) Control Diet (% w/w)	Zn(−) Diet (% w/w)
16:0	11.20 ± 0.11	11.57 ± 0.34
16:1	0.17 ± 0.01	0.18 ± 0.02
17:0	0.10 ± 0.01	0.11 ± 0.04
17:1	0.08 ± 0.03	0.07 ± 0.01
18:0	2.26 ± 0.04	2.31 ± 0.05
18:1 *n*-9	28.31 ± 0.15	28.42 ± 0.16
18:2 *n*-6	55.55 ± 0.22	55.19 ± 0.18
18:3 *n*-3	1.25 ± 0.03	1.28 ± 0.02
20:3 *n*-6	0.49 ± 0.06	0.43 ± 0.01
20:1 *n*-9	0.41 ± 0.01	0.42 ± 0.02

* Modified NRC [44] purified chicken diets were provided by Dyets Inc. (Bethlehem, PA, USA), (Zn(+), Zn adequate control diet: 135251, Zn(−), Zn deficient diet: 135252). ** Determination of dietary fatty acid composition is described in the Materials and Methods section.

Zinc intakes were calculated from feed intakes and zinc concentration in the diets. At the end of the study (day 28), birds were euthanized by carbon dioxide exposure. The digestive tracts (colon and small intestine) and liver were quickly removed from the carcass and separated into various sections for tissue analysis (~1–2 cm; ~2–3 g was taken from small intestine and liver, respectively). The samples were immediately frozen in liquid nitrogen, and then stored in a −80 °C freezer until analysis. All animal protocols were approved by the Cornell University Institutional Animal Care and Use committee.

### 2.2. Determination of Serum, Nail and Feather Zinc Content

Blood samples were collected weekly from the wing vein (*n* = 12, ~100 μL) using micro-hematocrit heparinized capillary tubes (Fisher Scientific, Pittsburgh, PA, USA). Samples were collected in the morning following an 8 h overnight fast. Nail and feather samples were collected on day 28 of the experiment (*n* = 12, ~1–2 g). Serum, nail and feather zinc concentrations were determined by an inductively-coupled argon-plasma/atomic emission spectrophotometer (ICAP 61E Thermal Jarrell Ash Trace Analyzer, Jarrell Ash Co., Franklin, MA, USA) following wet ashing.

### 2.3. Isolation of Total RNA

Total RNA was extracted from 30 mg of duodenal (proximal duodenum, *n* *=* 9) and liver tissues (*n* *=* 9) using Qiagen RNeasy Mini Kit (Qiagen Inc., Valencia, CA, USA) according to the manufacturer’s protocol. All steps were carried out under RNase free conditions. RNA was quantified by absorbance at 260–280 nm. Integrity of the 28S and 18S rRNA was verified by 1.5% agarose gel electrophoresis followed by ethidium bromide staining.

### 2.4. Gene Expression Analysis

As previously described [45,46,47,48], PCR was carried out with primers chosen from the fragments of chicken duodenal and hepatic tissues. Tissue-specific 18S rRNA was used to normalize the results. All PCR products were separated by electrophoresis on 2% agarose gels, stained with ethidium bromide, and quantified using the Quantity One 1-D analysis software (Bio-Rad, Hercules, CA, USA). Table 3 represents the totality of genes assessed in this study.

### 2.5. Fatty Acid Analysis of Erythrocytes and Experimental Diets

Blood samples were centrifuged at ~2000 *g* for 10–15 min at room temperature to fractionate whole blood. Total lipids were then extracted from red blood cells and experimental diets according to a modified Bligh and Dyer method [49]. Fatty acid methyl esters (FAMEs) were prepared using 14% boron trifluoride in methanol (Sigma Chemical, St. Louis, MO, USA). Butylated hydroxytoluene was added to methanol as an antioxidant. Heptadecanoic acid (Sigma Chemical, St Louis, MO, USA) in chloroform was used as an internal standard. FAME analyses were performed using a Hewlett Packard 5890 Gas Chromatograph (GC) (GMI Inc., Ramsey, MN, USA) with a flame ionization detector (FID) (GMI Inc., Ramsey, MN, USA). A BPX-70 column (25 m × 0.22 mm × 0.25 μm, SGE, Austin, TX, USA) was used for the analysis with H_2_ as the carrier gas. FAME identities were determined by a chemical ionization (CI) mass spectrometry (MS), using a Varian Star 3400 GC (Varian Inc., Walnut Creek, CA, USA) coupled to a Varian Saturn 2000 Ion Trap MS (Varian Inc., Walnut Creek, CA, USA). FAME identities were based on GC retention time of each substance and its CI mass spectra. An equal weight FAME mixture (68A; Nuchek Prep, Elysian, MN, USA) was used to calculate response factors. Fatty acid (FA) levels are expressed as weight % of total FA (% w/w).

**Table 3:** Measured genes (*Gallus gallus*) and tissue-specific 18S rRNA from mRNA.

Analyte	Organ	Forward Primer (5′→3′) (Nucleotide Position)	Reverse Primer (5′→3′) (Nucleotide Position)	Length	GI Identifier
				(Base Pairs)	
ZnT1	Intestine	CCTCCAGACAACCTTTGGTG (64–83)	TACTGATCTGCAAACCTTGCCA (133–112)	69	54109718
ZnT5	Intestine	TCGTGGAGGCTGTCATTCAC (1657–1676)	TGCAGATCTTTCTCCTGTTCGT (2016–1995)	359	56555150
ZnT7	Intestine	GGCGTCTGGAGTAACAGCTT (166–185)	GTGAATGCCCATGACCTCCA (502–483)	336	56555152
ZIP6	Intestine	TTGTGGAATCATCCCAGGGC (549–568)	GCTCATTCGCATCTCTCCGA (929–909)	380	66735072
ZIP9	Intestine	TTATTCCCCTGGCCGTGAAC (68–87)	CCAATGCGAAGACCAGCAAG (643–624)	575	237874618
TNF-α	Liver	CATTTGGAAGCAGCGTTCGG (48–67)	GACAGGGTAGGGGTGAGGAT (249–230)	202	53854909
IL-1β	Liver	CCTCCAGCCAGAAAGTGAGG (431–450)	TTGTAGCCCTTGATGCCCAG (539–520)	109	88702685
IL-6	Liver	AACAACCTCAACCTGCCCAA (338–357)	AGGTCTGAAAGGCGAACAGG (449–430)	112	302315692
NF-κB	Liver	GGATGGTCTGTTCCTGAAGA (1682–1702)	ACCTCTGCCTGCTTTGTGAT (1981–1961)	300	2130627
AP	Intestine	GAATGAGGGCTTTGCCTCCT (1245–1264)	GAAGTTGCTGTTGGTGGCTG (1854–1835)	610	45382360
SI	Intestine	CAGATCTCAGCCCGTCTTCC (237–256)	CCAGAATGCCACCGGTAACT (519–500)	282	2246388
Na + K + ATPase	Intestine	CTGAGGGCAACGAAACAGTG (104–123)	ATCCCTCGGGTTGACCTCC (177–159)	74	14330321
SGLT-1	Intestine	GTGGAATGCCTTGGAGGGTA (3–22)	GCTTCCTCAGATACTCCGGC (123–104)	121	8346783
MT4	Intestine	ACCCGAACTGAACCATGGAC (36–55)	TTTTCGTGGTCCCTGTCACC (312–293)	277	46048710
Δ^6^ desaturase	Liver	ACATGAACAGAGGAAGCGGG (780–799)	TCTGGATCTCCTCCCAGGTG (1754–1735)	975	261865208
DMT-1	Intestine	TTCCTCCTCAACAACGTCGG (1755–1774)	TCCCAATGCCATCCCAGTTC (1908–1889)	154	206597489
18S rRNA	Intestine, Liver	CGATGCTCTTAACTGAGT (1251–1269)	CAGCTTTGCAACCATACTC (1550–1531)	300	7262899

### 2.6. Determination of Phytic Acid Concentration in the Diet Samples

Dietary phytic acid (phytate)/total phosphorus was measured as phosphorus released by phytase and alkaline phosphatase, following the kit manufacturer’s instructions (five replicates per diet). The total phosphate released is measured using a modified colorimetric method and given as grams phosphorus per 100 g of sample material (K-PHYT 12/12, Magazyme International, Bray, Ireland).

### 2.7. Statistical Analysis

Results were analyzed by ANOVA using the general linear models procedure of JMP software (SAS Institute Inc., Cary, NC, USA). Differences between treatments were compared by Tukey’s test and values were considered statistically different at *p* < 0.05. Mixed effect model test between serum zinc (fixed effect) and bird group (random effect) was performed to elucidate a possible correlation between serum Zn and LA:DGLA ratio. The data are presented as least square means with their standard error of the mean.

## 3. Results

Diets were similar in all 16 to 20 carbon fatty acids (three replicates per diet, Table 2). Further, both the zinc adequeate and zinc deficient diets contained no detectable amounts of phytic acid (five replicates per diet, Table 1, kit detection limit 40 mg phytic acid/100 g sample).

Body weight, feed consumption, and zinc intake were consistently higher in the Zn(+) *versus* Zn(−) birds on days 7, 14, 21, and 28 (*n* = 12, *p* < 0.05, Table 4). Further, at each time point, serum zinc concentration for the Zn(+) control bird group was significantly higher than the Zn(−) bird group (*n* = 12, *p* < 0.05, Table 4).

**Figure 2:**
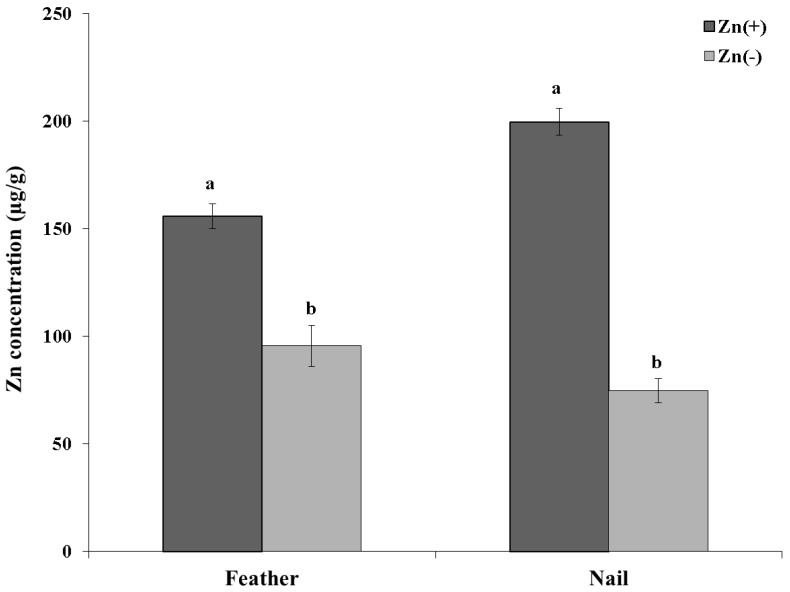
Chicken feather and nail zinc concentrations (day 28). ^a,b^ Within each parameter, means without a common letter are statistically significant (means ± SEM, *n* *=* 12, *p* < 0.05).

**Figure 3:**
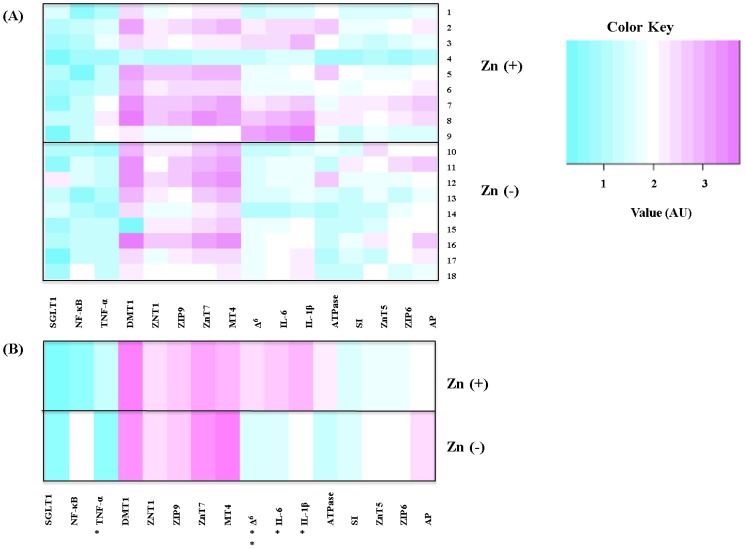
Intestinal (duodenal) and hepatic mRNA expression of zinc-related genes (day 28); changes in mRNA expression are shown relative to expression of 18S rRNA in arbitrary units (AU, *n* *=* 9). (**A**) Effect of zinc deficiency on gene expression. Treatment groups are represented as Zn(+) control: birds 1–9, and Zn(−): birds 10–18. Treatment groups are represented on the *y*-axis, and zinc related genes on the *x*-axis. (**B**) Mean effect of zinc deficiency on gene expression (* *p* < 0.05, ** *p* < 0.001).

Zinc concentration in both tissues (nail and feather) were increased in the Zn(+) group *versus* the Zn(−) group (day 28, *n* *=* 12, *p* < 0.05, Figure 2). Gene expression analysis of the intestinal tissue, with results reported relative to 18S rRNA, revealed a higher mean (AU) for TNF-α, IL-1β, and IL-6 in the Zn(+) *versus* Zn(−) group (*n* = 9, *p* < 0.05). Analysis of hepatic tissue for Δ^6^ desaturase relative to hepatic 18S rRNA showed significant differences between groups (*n* = 9, *p* < 0.001), with a higher mean (AU) value for Zn(+) *versus* Zn(−) group. No other assessed parameter (*zinc and metal transporters*: ZnT1, ZnT5, ZnT7, ZIP6, ZIP9, DMT-1; *transcription factor*: NF-κB; *brush border enzymes*: aminopeptidase, sucrase-isomaltase, Na^+^K^+^ATPase, SGLT-1; *binding protein*: MT4) was significantly different between the Zn(+) and Zn(−) groups (*n* = 9, *p* > 0.05, Figure 3A shows gene expression, Figure 3B shows mean difference per treatment).

**Figure 4:**
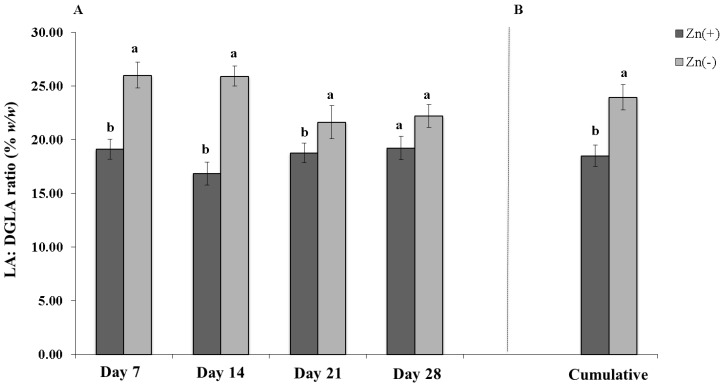
Chicken LA:DGLA ratio of Zn(+) control and Zn(−) groups. The LA:DGLA ratio is expressed as mass percent (% w/w). (**A**) Weekly chicken LA:DGLA ratio of Zn(+) and Zn(−) treatment groups (*n* *=* 12). ^a,b^ Within a time point, means without a common letter are statistically significant (*p* < 0.05). (**B**) Cumulative differences in LA:DGLA ratio between Zn(+) control and Zn(−) and treatment groups (*n* = 12). ^a,b^ Means without a common letter are significantly different (*p* < 0.001).

As for the LA:DGLA biomarker, the % w/w ratio was significantly elevated (*n* = 12, *p* < 0.05) in the Zn(−) group on days 7, 14, and 21 but not significantly different on day 28 (*p* = 0.0588) (Figure 4A). Overall, the biomarker was significantly different in reporting cumulative % w/w LA:DGLA ratio between the two groups, with the Zn(−) having a higher mean ratio (*n* *=* 12, *p* < 0.001, Figure 4B). The mixed effect model indicated a slight inverse trend between serum zinc and LA:DGLA (*m* = −0.18, *t*-ratio = −0.16 on 40.28 degrees of freedom, *p* = 0.1349).

**Table 4:** Body weight, feed consumption, zinc intake, and serum zinc concentrations in chickens fed a Zn(+) control and Zn(−) diets from day 0 to 28 *.

Treatment	Day 0	Day 7	Day 14	Day 21	Day 28
**Body weight** (g)					
Zn(+)	39.5 ^a^ ± 1.1	83.3 ^a^ ± 2.7	154.6 ^a^ ± 8.9	293.1 ^a^ ± 24.9	482.5 ^a^ ± 44.2
Zn(−)	38.8 ^a^ ± 1.2	72.1 ^b^ ± 2.7	106.9 ^b^ ± 5.3	132.8 ^b^ ± 6.6	147.7 ^b^ ± 14.4
**Feed consumption** (kg/day) **					
Zn(+)	−	0.152 ^a^ ± 0.007	0.194 ^a^ ± 0.009	0.253 ^a^ ± 0.013	0.329 ^a^ ± 0.016
Zn(−)	−	0.131 ^b^ ± 0.007	0.143 ^b^ ± 0.007	0.130 ^b^ ± 0.007	0.122 ^b^ ± 0.006
**Zinc intake** (g) ***					
Zn(+)	−	7.4 ^a^ ± 0.5	19.1 ^a^ ± 0.95	37.2 ^a^ ± 1.9	64.6 ^a^ ± 3.2
Zn(−)	−	0.40 ^b^ ± 0.03	0.84 ^b^ ± 0.04	1.1 ^b^ ± 0.06	1.4 ^b^ ± 0.07
**Serum zinc concentration** (μg/g)					
Zn(+)	−	3.42 ^a^ ± 0.19	4.66 ^a^ ± 0.35	4.73 ^a^ ± 0.25	4.49 ^a^ ± 0.23
Zn(−)	−	2.87 ^b^ ± 0.17	3.59 ^b^ ± 0.21	3.45 ^b^ ± 0.11	3.39 ^b^ ± 0.11

* Values are means ± SEM. ** Values are mean daily feed intakes for the seven days preceding the day designated in the column heading. *** Values are cumulative weekly from day 0. ^a,b^ Within a column and for each parameter, means without a common letter are significantly different (*n* = 12, *p* < 0.05).

## 4. Discussion

This study represents the initial steps in evaluating the LA:DGLA ratio as a biomarker of zinc status *in vivo*. Previously, the broiler chicken has been used for nutritional research and has been shown to be an accurate animal to use in iron bioavailability studies, as chicks respond quickly to malnutrition, and their micronutrient deficient phenotypes include poor iron status, growth stunting, and organ hypertrophy. Further this model agrees well with human *in vitro* cell line results [32,33,34,35]. In addition, the erythrocyte fatty acid composition is similar between birds and mammals [36].

Clearly, additional dietary intervention trials are needed to fully characterize the usefulness of this biomarker in relation to zinc status and zinc bioavailability over time. However, the initial results are promising as they demonstrate that dietary zinc concentration alone altered the LA:DGLA ratio, and that a significant and measurable difference for LA:DGLA was evident within the first week of zinc deprivation. Therefore, this ratio may be a sensitive and specific marker of zinc nutriture over an extended period of time.

In the present study, body weight, zinc intake, and serum zinc appear to be accurate markers of zinc deficiency, as they responded to zinc depletion in a dose-dependent fashion. Feather and nail zinc concentrations seem to be efficacious biomarkers of zinc status, as well. These results are in agreement with previous studies in humans which have found serum [50,51], hair and nail zinc concentration [22,52] reflective of dietary zinc intake.

In regard to serum zinc, Lowe *et al*. [22] have found that factors unrelated to zinc status or depletion, such as host infection, inflammation, time of measurement, sample handling, and many other parameters may disrupt the sensitivity of serum zinc, hence its sensitivity may be inconsistent.

The relative increase in gene expression of the cytokines TNF-α, IL-1β, and IL-6 in the Zn(+) group is in agreement with previous *in vitro* and *in vivo* studies where expression of various cytokines, especially TNF-α and IL-6, was downregulated in zinc deficient birds due to the dependency of the immune system on zinc [53,54]. The increase in hepatic Δ^6^ desaturase expression in the Zn(+) group was also expected, since zinc is an essential cofactor for the Δ^6^ desaturase enzyme. As such, zinc deficiency would impede functioning and gene expression of hepatic Δ^6^ desaturase. This observation is in accordance with previous studies [55,56].

In relation to the other zinc-related genes that were measured, previous studies have documented that even mild zinc deficiency alters many of the parameters we assessed (e.g., brush border enzymes [19], inflammatory cytokines [57,58], transporters and binding proteins) [59,60,61]. These observations are usually seen in subjects under zinc deficiency conditions and over an extended period of time. Thus, there is no question that zinc deficiency induces pathophysiological and morphological changes within the host. Rather, we suggest the possiblity that these mRNA gene expression biomarkers may not be sensitive enough to reveal differences in zinc status of deficient birds in a relativeley short feeding trial. Further, compensatory mechanisms (adaptation) by the animals to the zinc deficient diet may help to explain the lack of observational differences between many of the zinc-dependent mRNA parameters; as was previously demonstrated *in vivo* with the expression of DMT1 and under dietary iron deficient conditions [47]. Additional studies are required to elucidate the involvement of dietary zinc in the mechanism of action of zinc-dependent proteins and enzymes, and whether they may be responsive to dietary zinc manipulation over longer periods of time.

The results of the LA:DGLA ratio indicate that it is sensitive to changes in supplemental zinc intake. Previously, estimates by Maret *et al.* suggest zinc deficiency affects more than 25% of the world’s population, and poses a serious risk to public health [62]. A major cause of this deficiency is believed to be insufficient dietary zinc intake [62]. However, despite such a large number of people who are affected by the pathophysiological repercussions of inadequate zinc intake, development of reliable markers to assess such deficiency is still needed [63,64]. Although current indices of zinc status may be able to detect relatively large differences in zinc status, most are not sensitive enough to quantify changes in and between deficient states on the basis of dietary zinc intake [64,65]. The development of sensitive zinc biomarkers, thus, is key as suggested by World Health Organization reports [65]. In the current study, our results suggest the erythrocyte LA:DGLA ratio may be a sensitive tool to investigate the effects of *in vivo* dietary zinc manipulation under controlled dietary and environmental conditions. Further, an additional value of this biomarker is that it can be used to assess outcomes of changing levels of dietary zinc rapidly, as demonstrated in the current study where significant differences between groups occurred within seven days. Since mild to moderate zinc deficiency does not usually present with specific organ pathologies, clinical symptoms, if present, generally go undiagnosed [63]. Hence, this biomarker could possibly be used to detect early-stage zinc deficiency before the onset of symptoms and the progression to a more serious disease state. In addition, lack of consensus surrounding the reccomendation for zinc intake stems from the difficulties in obtaining a dependable indicator of zinc status [63].

Future feeding trials are now warranted to assess LA:DGLA sensitivity in a variety of new study parameters, such as amongst treatment groups with various levels of zinc deficiency and during an extended period of time, as well as elucidate Δ^6^ desaturase kinetics. In addition, future studies may also clarify any potential LA:DGLA limitations such as whether similar effects may be observed in mammals. We suggest that the continued development of this biomarker may aid in its application as a sensitive biological indicator of zinc status.

## 5. Conclusions

This study has explored the implementation of an easily measured, potential zinc biomarker pertaining to erythrocyte fatty acid composition, the LA:DGLA ratio. These data confirm the responsiveness of the erythrocyte LA:DGLA ratio to dietary zinc manipulation. Among a panel of purported zinc biomarkers, this ratio appears to be sensitive to dietary zinc depletion between bird groups over time. These data serve as a justification for further feeding trials which build on our results, especially those in which a diet more representative of the target zinc-deficient population is used.
